# Lymphome primitif laryngé: à propos d’un cas

**DOI:** 10.11604/pamj.2024.47.161.41032

**Published:** 2024-04-03

**Authors:** Madiha Chalout, Anouar Bouhlala, Oumaima El Masfioui, Naouar Ouattassi, Najib Benmansour, Mohamed Noureddine El Amine El Alami

**Affiliations:** 1 Université Sidi Mohamed Ben Abdellah, Faculté de Médecine et de Pharmacie de Fès, Centre Hospitalier Universitaire HASSAN II, Fès, Service Oto-Rhino-Laryngologie et Chirurgie Cervico-Faciale, Fès, Maroc

**Keywords:** Larynx, lymphome, laryngoscopie, chimiothérapie, cas clinique, Larynx, lymphoma, laryngoscopy, chemotherapy, case report

## Abstract

Le lymphome primitif du larynx, une entité rare, constitue moins de 1% de tous les cancers laryngés. Son traitement dépend de son stade et de sa gravité. Nous présentons le cas exceptionnel d´une femme de 64 ans, non-fumeuse, souffrant d´une dysphagie aux solides et d´une sensation de corps étranger. Une laryngoscopie et des biopsies ont révélé une tumeur polyploïde sur le repli ary-épiglottique gauche, confirmée comme un lymphome malin non hodgkinien B diffus à grandes cellules. La patiente a bénéficié d´une chimiothérapie suivie d´une radiothérapie, avec une amélioration notable sur les 2 années de suivi, sans récidive locale. En raison de sa rareté et de la variété des symptômes, la prise en charge optimale de ce type de cancer reste controversée, nécessitant une approche diagnostique et thérapeutique spécifique, ce qui en fait un cas intéressant à publier.

## Abstract (en)

Primary laryngeal lymphoma is rare, accounting for less than 1% of all laryngeal cancers. Treatment depends on the stage and severity of the disease. We here report the exceptional case of a 64-year-old woman, non-smoker, suffering from dysphagia for solids and a foreign body sensation. Laryngoscopy and biopsies revealed polyploid tumor of the left epiglottic fold. The diagnosis of diffuse large B-cell lymphoma was made. The patient underwent chemotherapy followed by radiotherapy, with significant improvement at 2-year follow-up, with no local recurrence. Due to the rarity of this disease and the variety of symptoms, the optimal management strategy for this type of cancer is controversial, requiring a specific diagnostic and therapeutic approach.

## Introduction

Le lymphome non hodgkinien (LNH) primitif survient le plus souvent dans le tractus gastro-intestinal, son apparition primitive au niveau du larynx est exceptionnelle, représentant moins de 1% de toutes les tumeurs du larynx [1,2]. Bien que les carcinomes épidermoïdes constituent 90% des tumeurs laryngées, le lymphome laryngé, notamment s´il affecte la région supra-glottique, est un diagnostic différentiel important qui se traite par une chimio-radiothérapie au lieu d´une chirurgie [3]. Nous rapportons à travers notre observation un cas exceptionnel d´un lymphome non hodgkinien primitif au niveau de l´étage supra-glottique.

## Patient et observation

**Information de la patiente**: une femme au foyer âgée de 64 ans, sans antécédents pathologiques notables, a consulté pour une dysphagie aux solides accompagnée d´une sensation de corps étranger pharyngé évoluant depuis un mois, non améliorées par les traitements symptomatiques. Elle ne présentait ni difficultés respiratoires ni dysphonie. Son état général était stable et elle ne présentait pas de fièvre.

**Figure 1:**
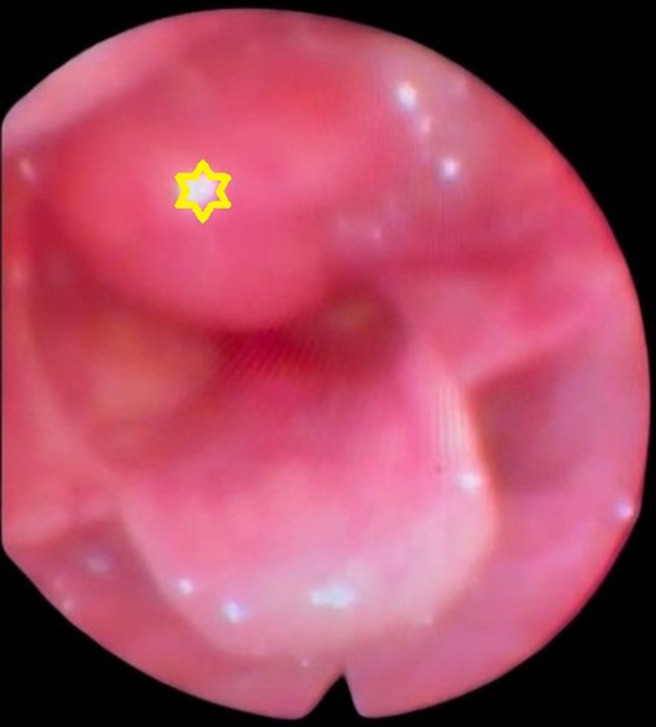
nasofibroscopie; une masse polyploïde du repli ary-épiglottique gauche

**Résultats cliniques**: à l´examen clinique, aucune anomalie ni adénopathie périphérique n´ont été constatées. La nasofibroscopie avait montré une tumeur polyploïde rosée avec une surface lisse et une muqueuse saine, localisée sur le repli ary-épiglottique gauche et son pédicule attaché dans la région rétro-crico-aryténoïdienne gauche (Figure 1).

**Figure 2:**
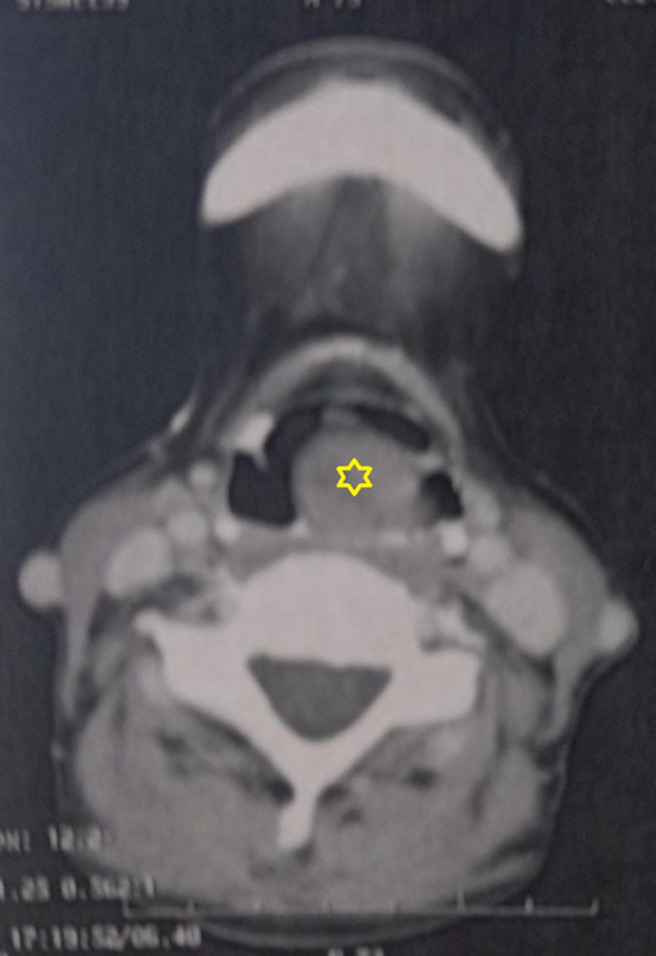
scanner cervico-thoracique en coupe axiale: une masse tissulaire homogène supra glottique

**Figure 3:**
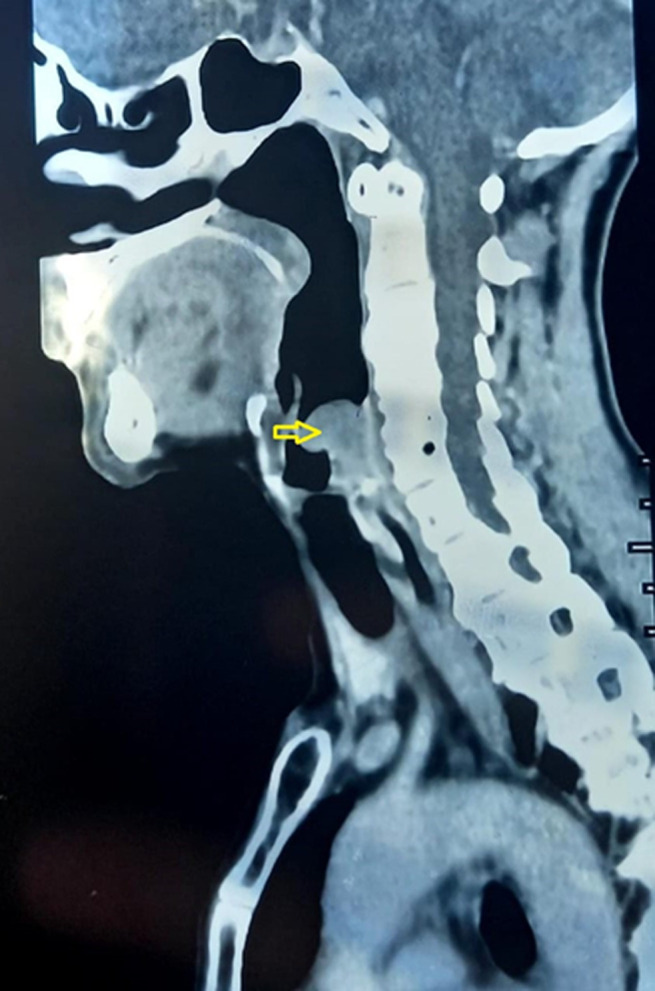
scanner cervico-thoracique en coupe sagittale: extension de la tumeur vers la paroi postérieure de l´hypo pharynx

**Figure 4:**
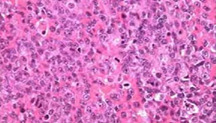
examen anatomopathologique: lymphome non hodgkinien primitif type B du larynx

**Évaluation diagnostique**: la tomodensitométrie cervico-thoracique (Figure 2, Figure 3) a révélé la présence d´une masse polyploïde bien délimitée, de densité tissulaire homogène qui se rehausse en plage après injection de produit de contraste, localisée au niveau de la paroi postérieure de l´hypo pharynx. Elle s´étend en avant vers l´étage supra glottique et entre en contact avec la face laryngée de l´épiglotte. Une laryngoscopie directe sous anesthésie générale avec des biopsies multiples a objectivé la présence d´une masse polyploïde rosée au niveau supra-glottique. L´analyse histologique a révélé la présence d´une infiltration de grandes cellules lymphoïdes caractérisées par des contours nucléaires irréguliers et la présence de multiples nucléoles proéminents (Figure 4). En immunohistochimie, les cellules tumorales présentent une expression positive pour le CD20, tandis qu´elles ne présentent pas d´expression pour le CD5, le CD7, le CD23, le CD10, la cycline D1, le LEF1 et le Bcl6. Des plasmocytes dispersés ont été identifiés, préservent le CD138, mais ils sont présents en quantité limitée. L´indice de prolifération, évalué à l´aide du marqueur Ki67, est affiché à 25%. Sur la base de ces résultats, le diagnostic d´un lymphome B de bas grade, compatible avec un lymphome de la zone marginale a été établi. Les résultats du bilan biologique étaient normaux, comprenant une formule de numération sanguine, des marqueurs d´inflammation et la mesure de la LDH. Les examens d´extension, y compris une TDM thoraco-abdomino-pelvienne et une biopsie ostéo-médullaire, n´ont révélé aucune anomalie, ce qui a permis de déterminer le stade de la maladie: IEBa selon la classification d´Ann Arbor.

**Intervention thérapeutique**: la patiente a reçu quatre cures de chimiothérapie type R-CEOP, qui consistent en un mélange de rituximab et de divers médicaments de produits, comprenant le cyclophosphamide, l´étoposide, la vincristine et la prednisone. Après chaque cycle, des intervalles appropriés ont été respectés, suivis d´une radiothérapie. La radiothérapie a été administrée sur une période de quatre semaines, avec une dose totale de 40 Gy. Elle a été répartie en cinq séances de 2 Gy par semaine, ciblant le site tumoral ainsi que les ganglions lymphatiques adjacents, y compris le médiastin supérieur.

**Suivi et résultats**: les effets secondaires du traitement étaient minimes. Après trois mois de traitement, la dysphagie avait complètement disparu. Les examens endoscopiques et tomodensitométriques de contrôle effectués six mois plus tard ont révélé des résultats normaux. L´évolution était favorable, sans aucun signe de récidive locale. La période de suivi s´étend sur deux ans.

**Perspectives de la patiente**: la patiente a affirmé que la dysphagie s´était atténuée avec tous les autres symptômes, notamment une amélioration significative de sa déglutition.

**Consentement éclairé**: la patiente a compris les conditions, l´importance des différents tests et des étapes de la prise en charge diagnostique, du traitement médical et du suivi post thérapeutique, et a consenti aux différentes étapes de la prise en charge et de la publication. La patiente a expressément consenti à la publication en donnant sa signature écrite sur le formulaire de consentement éclairé.

## Discussion

Le lymphome non hodgkinien primitif est fréquemment observé dans les sinus para nasaux, les glandes salivaires et la glande thyroïde dans la région de la tête et du cou [3]. En revanche, qu´il est très rare au niveau du larynx, représentant moins de 1% de tous les cancers laryngés, avec moins de 100 cas documentés. Cette rareté est due à la faible présence de tissu lymphoïde dans le larynx par rapport à d´autres parties des voies respiratoires. L´âge moyen au diagnostic est autour de 70 ans avec des extrêmes allant de 4 à 81 ans. Le rapport sex-ratio hommes-femmes variait d´une série à l´autre [4,5]. Les symptômes du lymphome malin non hodgkinien (LMNH) varient en fonction de sa localisation: une dysphonie, une dysphagie, une sensation d´un corps étranger pharyngé, des bruits respiratoires anormaux (stridor), ainsi que des symptômes généraux tels que perte de poids, sueurs nocturnes et fièvre. La détresse respiratoire est rare dans ces cas. Selon la synthèse des études disponibles, il est indiqué que tous les sites du larynx peuvent être touchés, mais la région supra-glottique est la plus courante, suivie de la région glottique. Les régions para-glottiques et sous-glottiques sont moins souvent touchées [6].

Certains signes d´imagerie suggèrent la possibilité d´un lymphome, notamment une grande lésion supra-glottique présentant un rehaussement uniforme de la couche sous-muqueuse, typique du lymphome plutôt que du carcinome épidermoïde [6,7]. Le lymphome laryngé se propage vers les cartilages du larynx, l´hypopharynx et l´oropharynx, sans calcifications observées. Macroscopiquement, ces tumeurs se manifestent sous forme de masse sous-muqueuse ou de tumeur polyploïde, avec une surface lisse et une couleur blanc-grisâtre [7]. Pour confirmer le diagnostic, des échantillons de biopsie suffisamment représentatifs sont nécessaires pour une évaluation histologique précise. L´immunohistochimie permet de confirmer le phénotype des cellules malignes, B (marqué par la présence de CD20, CD19, CD22 et d´immunoglobulines de surface) ou T (marqué par la présence de CD2, CD3). Une analyse cytogénétique ou moléculaire peut compléter le diagnostic [8]. Selon la classification l´Organisation mondiale de la Santé (OMS), le lymphome non hodgkinien B représente 85% des cas, avec différentes sous-catégories telles que diffus à grandes cellules, folliculaire et du manteau, Ces sous-types ont été observés dans environ 50%,10% et 5% des cas respectivement [7,8].

Le traitement du lymphome varie selon son grade, son étendu et son stade. Les principales options thérapeutiques incluent la radiothérapie seule ou en combinaison avec la chimiothérapie [9]. Dans le cas de ces tumeurs, la radiothérapie est généralement la modalité de traitement principale. Elle permet d´obtenir une réponse complète prolongée chez 50 à 90% des patients atteints d´un lymphome de stade I ou de stade II localisé. Cependant, étant donné que la plupart des cas de lymphome sont de nature systémique, un protocole à base de CHOP (Cyclophosphamide, Hydroxydaunorubicine, Oncovin, Prednisone) avec ou sans rituximab selon le type histologique, joue un rôle important, en particulier dans les lymphomes de bas grade [8,9]. Les lymphomes non hodgkiniens primitifs du larynx ont un pronostic et un mode d´évolutions similaires aux lymphomes ganglionnaires (impliquant les ganglions sus et sous-diaphragmatiques, envahissant la moelle osseuse, etc.). La survie à 10-15 ans est estimée entre 50 et 60% [9].

## Conclusion

Malgré sa rareté, il est important de considérer le diagnostic d´un lymphome non hodgkinien primitif du larynx en présence de signes persistants, en particulier une masse sus-glottique, comme dans le cas de notre patiente, afin d´exclure un carcinome épidermoïde par une analyse anatomopathologique. Cela permet une adaptation thérapeutique appropriée et une amélioration du pronostic.
